# Wood Waste Valorization and Classification Approaches: A systematic review

**DOI:** 10.12688/openreseurope.18862.1

**Published:** 2025-01-10

**Authors:** AKRIVI KORBA, Lucyna Lekawska-Andrinopoulou, Kostas Chatziioannou, Georgios Tsimiklis, Angelos Amditis

**Affiliations:** 1 Institute of Communication and Computer Systems (ICCS), National Technical University of Athens, 9, Iroon Politechniou Str., Zografou Campus, Athens, 15773, Greece

**Keywords:** Wood Waste Valorization, Wood Waste Classification, Systematic Review, Circular Economy

## Abstract

This systematic literature review delves into various wood waste valorization and classification approaches, aiming to evaluate their efficacy in fostering sustainable wood resource management while enhancing the economic value of wood waste. By synthesizing findings from a diverse array of research studies, the review highlights the multifaceted nature of wood waste valorization, emphasizing the critical role of sorting and separation technologies in ensuring high-quality recovery of materials. It also identifies the wood classification practices in Europe, which are crucial for creating a harmonized valorization framework that aligns technological advancements with regulatory standards. The analysis reveals that integrating these components—technologies, sorting methods, and classification practices can significantly improve the overall efficiency and effectiveness of wood waste management. Furthermore, the review identifies existing gaps in research and practice, providing actionable recommendations for stakeholders aiming to optimize wood valorization waste systems. These recommendations emphasize the necessity for a holistic approach and a clearly defined, comprehensive framework for wood valorization that considers all elements involved in the process. By addressing these areas, the review not only aims to contribute to the body of knowledge in wood waste valorization but also seeks to promote sustainable practices that benefit both the environment and the economy, paving the way for a more circular approach to wood resource utilization.

## Plain summary

This systematic literature review examines various approaches to wood waste valorization and classification, with the aim of evaluating their effectiveness in promoting sustainable wood resource management and increasing the economic value of wood waste. The review focuses on technologies for valorizing wood waste from construction, demolition, and furniture, with attention to treatment methods, key technologies like sorting and separation, and best practices, while also identifying challenges, opportunities, and research gaps that could support sustainable wood resource management and a circular economy.

A Systematic Literature Review (SLR) approach was employed to comprehensively assess valorization technologies for wood waste derived from construction, demolition, and furniture waste, following the methodology outlined by Harris *et al.* (2013). The review addresses the research question: What wood valorization technologies (for both pure and mixed treatment) are currently available, and what criteria should be considered when selecting a valorization route? To answer this question, contextual keywords were carefully analyzed and structured into a search query with three levels. A search string was developed using Boolean operators, and filters were applied based on article types and inclusion criteria. A total of 152 papers were identified from searches conducted in Science Direct and Scopus, following the PRISMA review technique to ensure transparency and repeatability.

The findings highlight the current state of research in wood valorization and classification, revealing significant gaps in the comprehensive exploration of technologies, sorting methods, and classification frameworks. The need for further development in integrating classification and valorization strategies to achieve sustainable wood management is emphasized. The strength of evidence is high, as despite the challenges posed by the limited number of scholarly papers, valuable and relevant insights were successfully drawn from the available literature. The refined search query navigated these constraints by effectively balancing specificity and breadth, revealing critical gaps in the field and providing a robust foundation for understanding current knowledge. This approach not only enhances the understanding of wood waste valorization but also helps inform future research directions.

In conclusion, wood valorization technologies have the potential to greatly improve resource efficiency, economic growth, and environmental sustainability by converting wood waste into valuable products. However, the successful implementation of these technologies requires addressing key challenges, such as sorting, separation, and harmonizing classification schemes. Looking ahead, a holistic approach is needed that integrates supply chain management, financial considerations, and Life Cycle Assessment (LCA) to optimize both the environmental and economic viability of valorization processes. A comprehensive framework that incorporates these elements will support sustainable practices and contribute to a circular economy within the wood industry, using this study as preliminary step.

## Introduction

### Background

The average European generates approximately 5 tons of waste annually, yet only 39% of this waste is recycled within the EU, highlighting a significant gap in waste management practices ( European Commission, 2020). A considerable portion of this waste consists of wood waste, including construction and demolition waste (CDW) accounting for over a third of total waste generated in the EU ( Eurostat, 2021). Additionally, another significant source of wood waste source is furniture sector. Around 10 million tons of furniture are discarded each year by businesses and consumers across EU countries, with the vast majority ending up in landfills or being incinerated, which contributes to environmental degradation ( European Commission, 2018). Both CDW and furniture wood waste streams contain significant amounts of fossil-based carbon, creating opportunities for recycling through innovative approaches. By harnessing novel technologies and methods, there is potential to reclaim this carbon and convert it into valuable resources, thereby promoting sustainability and reducing the environmental impact of wood waste. Effectively addressing these wood waste streams not only helps mitigate landfill use but also supports the transition to a circular economy, where materials are reused and repurposed, ultimately minimizing the demand for virgin resources.

Ongoing research into waste wood reuse and recycling has led to a myriad of approaches and technologies designed to optimize the valorization of wood waste; however, it is crucial to understand how to effectively manage wood waste based on its source matrix. Different types of wood waste, such as construction debris, industrial offcuts, and post-consumer products, exhibit varying characteristics that influence their potential for reuse and recycling. Consequently, tailoring valorization strategies to these specific contexts is essential for maximizing resource recovery and minimizing environmental impact. While numerous wood waste valorization processes exist, there is a pressing need for comprehensive research that summarizes and categorizes these methods, thereby providing a clearer picture of their efficacy and applicability ( Nunes & Figueiredo, 2020).

Furthermore, the lack of a structured and standardized framework for assessing these valorization processes complicates decision-making for stakeholders, making it challenging to evaluate which methods are most suitable for different types of wood waste. Establishing clear assessment criteria would not only facilitate better comparisons among technologies but also promote best practices and drive innovation in the sector. This gap underscores the importance of continued research efforts to develop cohesive framework that address these challenges, ultimately leading to more effective management and valorization of wood waste resources. By fostering a better understanding of source-specific characteristics and standardizing assessment methodologies, the industry can enhance its ability to implement sustainable and economically viable wood waste valorization practices.

This systematic review serves as a preparatory foundation for the development of a comprehensive wood valorization framework by synthesizing existing knowledge and identifying critical gaps in the current understanding of wood waste management technologies. By systematic analysis of diverse studies on wood valorization technologies, sorting techniques, and classification practices, the review highlights the complexities and interdependencies inherent in the valorization process. Additionally, the review elucidates the key criteria that should guide the selection of valorization routes, paving the way for a more structured and informed framework. By bringing together insights from various disciplines and practices, the systematic review facilitates the integration of innovative technologies, regulatory considerations, and sustainability goals, ultimately fostering a holistic approach to wood resource management. Such groundwork is essential for ensuring that the future framework is robust, adaptable, and capable of addressing the dynamic challenges associated with wood valorization, thereby enhancing both economic and environmental outcomes in the industry.

### Scope

This systematic review aims to evaluate the available approaches and technologies for valorizing wood waste from construction and demolition waste (CDW) and furniture waste, focusing on both pure and mixed treatment methods. It examines existing strategies and pathways, assessing their effectiveness in promoting sustainable wood resource management. The research identifies key components such as wood classification, sorting, and separation technologies, as well as valorization processes. It emphasizes best practices, challenges, and opportunities for improvement. Ultimately, this study seeks to guide future research, inform policy development, and support practical applications in wood waste valorization, contributing to the circular economy within the wood industry. By synthesizing current knowledge, it aims to pinpoint research gaps, showcase successful case studies, and advance research and practices in wood valorization.

## Methods

### Research protocol

This study employs a Systematic Literature Review (SLR) approach to provide a thorough review of valorization technologies for wood waste that comes from CDW and furniture waste, following the outline provided by Harris *et al.* (2013). A Systematic Literature Review (SLR) is a rigorous method of reviewing and synthesizing research on a specific topic. It follows a structured approach to ensure comprehensive coverage and minimize bias. An SLR provides a comprehensive overview of the current evidence, helping to inform decision-making and identify gaps for future research. The SLR is adapted to the field of information technology and adheres to Preferred Reporting Items for Systematic Reviews and Meta-Analyses (PRISMA) guidelines ( Moher *et al.*, 2009). The PRISMA methodology provides a structured approach to conducting systematic reviews. It consists of a 27-item checklist and a flow diagram, that aim to improve reporting transparency and quality. Following the PRISMA methodology allows researchers to improve the rigor and clarity of their systematic reviews, allowing them to make more valuable contributions to the field. Grey literature research was conducted using the customized Google search technique outlined by Godin *et al.* (2015), to effectively locate and retrieve relevant documents, reports and other non-peer-reviewed sources, that may provide valuable insights and data not captured in traditional academic databases.

### Eligibility criteria

In developing inclusion criteria for our systematic review on wood valorization, it was essential to be clear and specific to capture relevant and high-quality studies. The review initially aimed to explore existing wood waste valorization frameworks. This type of studies however was not identified in the scientific literature. Consequently, the focus was shifted towards the technologies involved in wood valorization and their categorization, while also defining the criteria useful for the novel wood waste valorization framework. This approach enables us to encompass a comprehensive range of relevant studies, thereby offering valuable insights into current practices, challenges, and opportunities in the field.

 In terms of content, study results must focus on the value of wood or wood-based materials, such as waste wood, byproducts, and residues. In addition, research should include a variety of valorization pathways, such as physical processes (e.g., wood composites and construction materials), biological processes (such as bioremediation and enzymatic treatments), energy recovery (such as biomass energy production), and chemical processes (e.g., conversion to biofuels or chemicals). Regarding the type of the articles, study findings may include peer-reviewed articles, conference papers, theses, and reports that employ experimental studies, case studies, reviews and meta-analyses, economic assessments and life cycle analyses.

Studies that did not specifically address the value of wood or wood-based materials were excluded. Study results that focused on unrelated materials (e.g., plastic, metal) or did not address any aspect of wood waste valorization were also excluded. Other exclusions were non-peer-reviewed articles (e.g., opinion pieces, editorials) documents in the form of book chapter, encyclopedia, editorial, conference abstract, discussion or mini review, documents that were not written in English, and duplicates publications or studies presenting overlapping data sets. These exclusion criteria help to narrow down the research findings to those that are relevant and of sufficient quality, ensuring that the systematic review captures valuable insights towards developing wood waste valorization framework.

### Search strategy

The growing demand for sustainable materials, accompanied by the need for effective waste management strategies, has reignited the interest in wood waste valorization. This process involves converting wood residues and low-value wood products into higher-value materials, chemicals, and energy, which contribute significantly to a circular economy ( Smith & Doe, 2022). Despite the potential benefits, current wood valorization approaches frequently lack standardization and comprehensive evaluation, resulting in several constraints and inefficiencies during implementation. Furthermore, the wide range of available technologies, processes, and methodologies, each one with the potential to be utilized in different contexts and applications, complicates understanding of best practices and their economic and environmental consequences.

This study comprises an evidence-based method to address the following research question (RQ):

*What wood (from CDW and furniture waste) valorization technologies (for pure and mixed treatment) are currently available, and what criteria to take into consideration when choosing valorization route?*

To answer this research question, the fundamental contextual keywords were meticulously analyzed and structured in a query consisting of three levels. We created a search string that consolidated keywords with Boolean operators, and we applied filters for the type of articles and the wordings per inclusion criteria. The structure of the query is shown in Table 1.

**Table 1.** Research Query.

Date of Search	Level	Keywords Synthesis
30May2024	Level 1	(''Wood Waste'') AND
	Level 2	(''Valorization'' OR ''Upcycling'' OR ''Classification'') AND
	Level 3	(''Process'' OR ''Approach'' OR ''Route'' OR ''Pathway'' OR ''Technology'')

The research query is structured across three distinct levels to explore wood waste valorization comprehensively. At Level 1, "Wood Waste" is the foundational concept, establishing the primary subject of inquiry. Level 2 broadens the focus by including terms such as "Valorization," "Upcycling," and "Classification," which reflect strategies for managing and utilizing wood waste effectively. Level 3 further refines the scope by incorporating terms like "Process," "Approach," "Route," "Pathway," and "Technology," which specify the mechanisms and methodologies used in transforming wood waste. This three-tiered structure ensures an in-depth investigation of both the theoretical and practical dimensions of wood waste valorization. To optimize the research query, keywords were iteratively refined, tested, and evaluated to ensure alignment with the research objectives.

### Study selection

**Figure 1.**
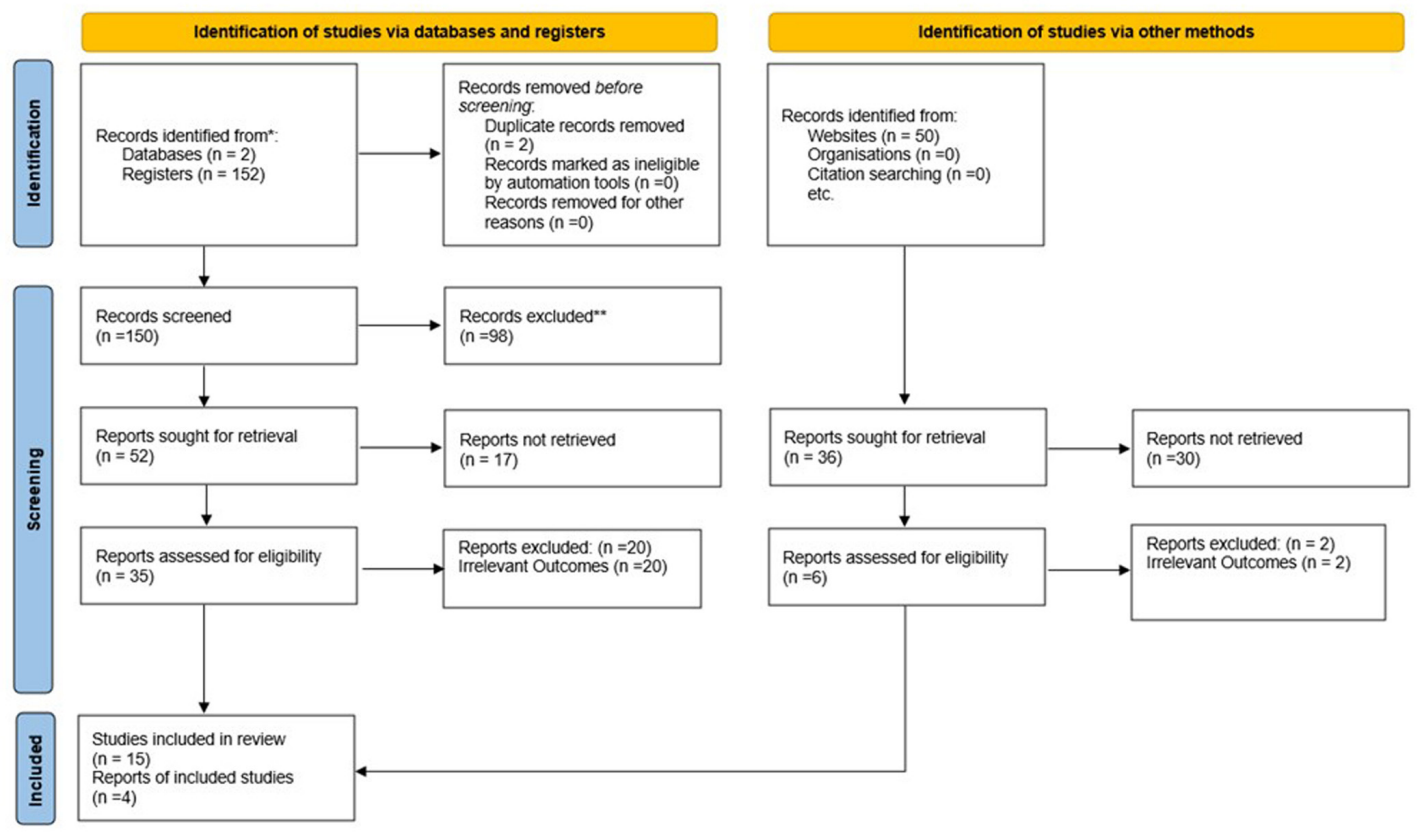
Identification of studies from databases (PRISMA methodology).

The query was initially run using both "Valorization" and "Valorisation" terms to capture all relevant data. This has resulted in 64 additional results. After eliminating duplicates, 150 articles were available for further screening based on the title, abstract, and keywords from ScienceDirect and Scopus databases. Figure 1 illustrates the study selection process following PRISMA guidelines to ensure transparency and reproducibility. Two independent reviewers evaluated each document to avoid potential bias and errors. Out of the 150 articles, one reviewer initially assessed 52 relevant papers, focusing on the inclusion criteria and relevance to wood waste valorization. From this review, 35 papers were deemed appropriate for further content analysis. Both reviewers then independently evaluated these papers, resulting in a final selection of 15 eligible studies for detailed assessment.

### Data extraction

The primary goal of this review was to capture comprehensive data on current wood valorization technologies and techniques. Data were extracted in four main categories:

1. Valorization Processes: Information on available wood valorization processes and their characteristics was compiled to provide insights into current methodologies.

2. Sorting and Separation Techniques: Data on wood sorting and separation methods were gathered, with an emphasis on how these techniques affect valorization efficiency.

3. Classification Decision-Tools: A dedicated set focused on tools for decision-making in wood classification, offering a systematic approach for categorizing wood based on its valorization potential.

4. Existing Classification Schemes: The final set examined existing classification schemes and relevant literature, emphasizing their role in standardizing wood valorization practices.

Each dataset was meticulously documented to ensure it aligned with the research objectives.

### Quality assessment

The quality and potential biases of the selected studies were meticulously evaluated to ensure reliable and valid findings in this review of wood valorization technologies. A structured assessment of study design and reporting standards was performed to confirm the reliability and applicability of the results. The PRISMA guidelines were adopted as a framework to enhance the transparency and rigor of the systematic review process.

It should be noted that a software tool was not utilized for the quality assessment due to the relatively small number of included studies. Instead, the evaluation was carried out by two independent researchers who rigorously assessed the quality of the studies. The inclusion criteria, defined by default to ensure high quality, included the use of trusted databases and highly cited journals, further guaranteeing the relevance and reliability of the selected studies.

### Data synthesis

Data synthesis was conducted on a final selection of 15 studies, supplemented by four additional documents from grey literature. The synthesis approach was qualitative, focusing on the extraction and interpretation of key insights rather than statistical or quantitative analysis. Data were categorized as outlined in the data extraction section, facilitating a structured approach to analysis.

Patterns across studies were analyzed to gauge confidence levels, with outcomes categorized based on the strength and reliability of the supporting evidence. Using narrative analysis, the findings were clustered by recurring themes, allowing for the identification of trends, patterns, and interpretative metrics within the data. This approach provided a comprehensive understanding of the diverse perspectives presented in the literature, integrating information across technological and environmental dimensions.

By emphasizing qualitative metrics, the analysis captured the nuances and context of each study, offering a richer interpretation of the implications for wood valorization. The findings, detailed in the Results and Discussion sections, highlight areas with strong evidence as well as those requiring further research, offering a holistic view of the potential impact of wood valorization technologies within the field.

## Results

### Overview of findings

This delineation of findings highlights not only the current state of knowledge in wood valorization and classification but also underscores the areas that require further exploration and development to achieve effective and sustainable wood management practices. In particular, the research findings, sourced from both academic and grey literature, can be categorized into four distinct groups: articles focused on wood valorization technologies, those addressing wood sorting and separation techniques, those presenting a wood classification frameworks or decision tools, or approach, and those discussion wood classification policies and regulations. Notably, only one paper was identified that discussed a wood waste classification decision tool. Additionally, three articles provided insights into sorting and separation techniques. The majority of the papers—constituting the most substantial category—focused on a variety of wood valorization methods and technologies; however, they did not adequately capture the breadth of these technologies.

To ensure confidence in the findings, assessments were conducted to evaluate the reliability and strength of the evidence supporting each outcome, highlighting the degree of certainty in the conclusions drawn from the review. Assessments were performed to gauge the reliability and robustness of the evidence for each outcome, strengthening confidence in the findings and ensuring that conclusions drawn are well-supported. A risk of bias assessment was conducted for each study category, examining reporting completeness, methodological rigor, and potential publication bias. Furthermore, results of all sensitivity analyses were conducted and are presented to assess the robustness of the synthesized findings. These analyses tested the influence of varying methodological choices and included examining the effects of study exclusion criteria, ensuring the stability and reliability of the synthesized conclusions 

Furthermore, it is important to note the differences in database outputs; the Science Direct database yielded publications primarily focused on wood sorting and separation and one publication presenting a wood classification decision tool, while Scopus predominantly provided articles related to wood valorization technologies and processes, which formed the foundation of our research. Additionally, grey literature contributed only articles pertaining to wood classification initiatives, policies, and regulations, further emphasizing the need for comprehensive research that bridges the gap between classification and valorization. Table 2 delineates the quantity of research articles examined within the framework of this systematic review, categorized by database and the proposed classification scheme.

**Table 2.** Research Results Overview-Wood valorization and classification with publication counts by database.

	Research Database		
	Science Direct	Scopus	Grey Literature
**Wood valorization**	3	8	0
**Wood Sorting and Separation**	3	0	0
**Wood Classification Decision-tool**	1	0	0
**Wood Classification Policies and Regulations**	0	0	4

### Wood valorization processes

**Figure 2.**
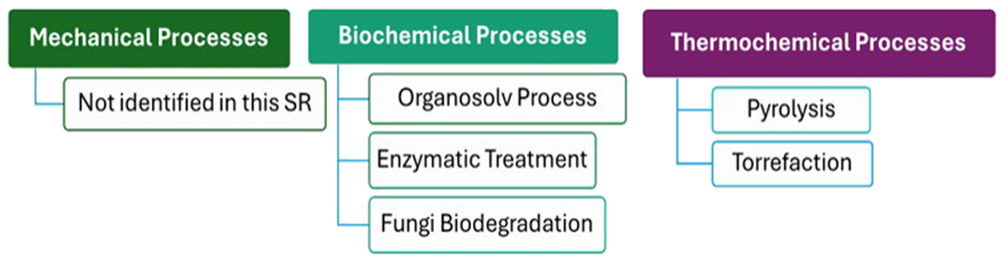
Classification of Wood Valorization Process identified in this systematic review.

The review's assessment of the scientific literature uncovered a total of 11 scientific papers focused on various wood valorization processes and technologies, with 3 sourced from Science Direct and 8 from Scopus. The identified literature can be categorized into two primary classifications of wood waste technologies: biochemical processes, and thermochemical processes. Biochemical processes include techniques like enzymatic treatment and remediation, leverage biological agents and chemical reactions to enhance the valorization of wood waste. Thermochemical processes, such as pyrolysis and torrefaction, utilize high temperatures to convert wood into energy-rich products, offering an effective means of waste disposal while generating valuable byproducts. Figure 2 illustrates all the valorization processes and technologies discovered during this study, which are classified as biochemical and thermochemical technologies.

***Biochemical processes.*** Biochemical processes or technologies involve organic solvents to break down lignocellulosic biomass, such as wood, into valuable chemical constituents, s garnered significant attention in recent research.

The organosolv process is a biochemical typically employs solvents like ethanol, methanol, or acetone, often in combination with water, to solubilize hemicellulose and cellulose while leaving behind lignin. The resulting products can be further refined into biofuels, biochemicals, and other high-value materials, contributing to a more sustainable and circular economy. Two scientific articles refer to the organosolv process a promising wood valorization technology that involves the use of organic solvents to break down lignocellulosic biomass, such as wood, into valuable chemical constituents The first article by Pazzaglia *et al.* (2023) examines wood waste sourced from a mechanical treatment plant that underwent organosolv treatment, resulting in cellulose pulp appropriate to produce containerboard. In the second paper conducted by Terzopoulou *et al.* (2022), organosolv lignin derived from beech wood was utilized as a filler within a poly (lactic acid) (PLA) matrix for the fabrication of composite materials. The organosolv process is particularly attractive due to its ability to operate under mild conditions and its potential for reducing environmental impacts compared to traditional methods. Additionally, the selective extraction of lignin enhances opportunities for its use in various applications, from adhesives to carbon fibers, making the organosolv process a key player in advancing wood valorization strategies.

Furthermore, two articles were identified that investigate distinct biochemical methods of wood treatment. These approaches not only enhance wood properties but also integrate biological systems into production processes, further supporting the transition towards sustainability. In particular, Charpentier-Alfaro *et al.* (2023) examine the application of fungi in wood treatment, emphasizing its potential advantages and underlying mechanisms. In contrast, Silber *et al.* (2023) concentrate on enzymatic treatment, specifically the production of insulation panels using fungal mycelium and lignocellulosic materials as substrates. This study details the enzymes derived from plants and organisms involved in wood synthesis and degradation, discussing their technical implementation in production processes such as additive manufacturing. By integrating biological systems into these technical processes, the research supports the industry's transition towards a circular economy.

Finally, there was one paper discovered, focusing on wood waste treatment, and specifically on effluents, by Salamat *et al.* (2018). The article reports on a straightforward and effective eco-friendly approach for the valorization of wood industry waste. The authors describe the development of adsorbent biomaterials derived from this waste, highlighting their potential environmental applications. This innovative strategy not only addresses waste management issues but also promotes sustainable practices by transforming by-products into valuable resources for wastewater treatment.

***Thermochemical processes.*** The utilization of pyrolysis as a treatment method for wood waste presents significant opportunities for sustainable waste management and resource recovery. This potential is reflected in our research, which explores the efficacy of pyrolysis in enhancing sustainable waste management and resource recovery practices. Haryanto *et al.* (2021) provide a comprehensive review of the pyrolysis process applied to wood wastes in Indonesia, highlighting its potential as an effective waste treatment strategy. The authors note that the pyrolysis of waste timber yields emissions comparable to those produced by clean wood, suggesting that this method can mitigate environmental impacts associated with waste disposal. Supporting this perspective, Sørmo *et al.* (2020) advocate for the consideration of pyrolysis as a viable treatment option for lightly contaminated organic waste. Together, these studies underscore the role of pyrolysis in promoting sustainable waste management practices while facilitating the valorization of wood residues.

Torrefaction might be perceived as a moderate kind of pyrolysis. Torrefaction is a thermal pretreatment process that involves heating biomass in an inert atmosphere at elevated temperatures, typically between 200 and 300 degrees Celsius, to improve its energy density, hydrophobicity, and combustion properties, thereby enhancing its suitability as a renewable energy source. Both processes involve the thermal degradation of biomass in an inert atmosphere, but torrefaction typically occurs at lower temperatures (between 200 and 300 degrees Celsius) and for shorter durations than traditional pyrolysis. While pyrolysis generally aims to produce bio-oils, gases, and char, torrefaction primarily focuses on improving the energy density and storage properties of biomass, making it more suitable for combustion and gasification processes. Cahyanti *et al.* (2021) demonstrate that torrefaction, a thermal pretreatment process, can enhance the properties of biomass as an energy source; their study specifically investigates the impact of various torrefaction operating parameters on the fuel characteristics of agricultural and wood waste.

In addition, the properties of various biomass residues were also identified and investigated as part of this research to evaluate their potential applications in energy production and environmental management. Specifically, two studies in literature delve into the properties of various biomass residues to evaluate their potential applications in energy production and environmental management. Saeed *et al.* (2022) investigated the explosion and flame propagation characteristics of typical Spruce-Pine-Fir residues obtained from a sawmill, providing insights into their safety and performance as fuel sources. In contrast, Ohenoja *et al.* (2019) focused on the fly ashes generated from the fluidized bed combustion of peat, wood, and waste materials, exploring whether these ashes could be modified through mechanical classification and grinding to meet relevant standards. Together, these studies contribute to a deeper understanding of biomass residues, highlighting their significance in advancing sustainable energy practices and waste management strategies.

Finally, another promising avenue identified in this study is the production of bioethanol from woody biomass, which underscores its potential as a viable renewable energy source with significant environmental sustainability benefits. The review by Hage *et al.* (2023) on bioethanol production from woody biomass aims to comprehensively compare recent bioconversion processes applied to woody substrates over the past five years, with a particular focus on thermomechanical pretreatments. Additionally, the review will address the outcomes of these individual steps, their implications for the overall bioconversion process, and their energetic considerations, thereby providing valuable insights into optimizing bioethanol production.

### Wood sorting and separation

The review's assessment of the scientific literature revealed 3 publications focused on the application of near-infrared (NIR) hyperspectral technology for waste extraction and classification, highlighting its potential as a transformative tool in these processes. NIR hyperspectral imaging offers a non-destructive and efficient method for analyzing the chemical and physical properties of materials, which is particularly advantageous in the context of waste management. In the first study, by Xiao *et al.* (2019), wood feedstock was sourced from the construction industry, emphasizing the technology's applicability in recycling construction and demolition debris. This study explored how NIR hyperspectral imaging can effectively differentiate between various wood types and grades, enhancing sorting efficiency and promoting better resource recovery. In the second paper by Mancini *et al.* (2018), the research shifted focus to different residues from the wood processing industry, demonstrating the versatility of NIR technology across diverse applications. By utilizing wood processing residues, this study illustrated how NIR hyperspectral imaging could aid in identifying valuable components within waste streams, ultimately contributing to more sustainable waste management practices. Collectively, these publications underscore the importance of NIR hyperspectral technology in advancing waste extraction and classification processes, paving the way for improved efficiency and effectiveness in wood waste valorization. The findings indicate a growing recognition of the technology's potential to support the circular economy by facilitating the recovery of useful materials from waste.

The third paper by Konstantinidis *et al.* (2023), is introducing an innovative deep learning multi-modal approach that leverages multiple parallel autoencoders to extract and analyze spatial-spectral information from both RGB and multi-spectral sensors. This advanced technique allows for the integration of data from different modalities, enabling a comprehensive understanding of the characteristics of various objects within the waste stream. By projecting the extracted features into a common latent space, the system can effectively represent complex relationships between the data points, facilitating a more nuanced interpretation of the information. Once the latent space representations are decoded, the model can accurately classify each object, determining its specific category based on the learned features. This classification process plays a crucial role in guiding the robotic sub-system, allowing it to make informed decisions about how to sort and process the waste materials. By incorporating this deep learning framework, the proposed system enhances the efficiency and accuracy of waste sorting operations, ultimately contributing to more effective waste management practices. The combination of RGB and multi-spectral data not only improves the robustness of the classification but also opens avenues for further research into the potential applications of multi-modal learning in various industrial contexts, paving the way for smarter and more adaptable waste management solutions.

### Wood classification decision-tool

Understanding and classifying wood waste based on its chemical composition can significantly enhance sorting processes and improve the overall utilization of this valuable resource. By adopting a chemical-based classification approach, it becomes possible to identify and recover essential components such as cellulose, lignin, and hemicellulose, which can be utilized in various applications ranging from biofuels to composite materials. In this context, Pazzaglia *et al.* introduced a comprehensive framework for wood classification that serves as a decision tool for determining the most suitable fate for wood waste based on its chemical composition. This methodology relies on the recognition of European Waste Catalogue (EWC) codes used by waste producers, which align with established European legislation. The systematic identification of EWC codes enables more precise categorization of wood waste, thereby facilitating targeted recycling and valorization strategies. Additionally, the framework is bolstered by a case study involving wood waste collected from a mechanical treatment plant in Perugia, Italy, which illustrates the practical application of the decision tool in a real-world setting.

### Wood classification policies and regulations

The contributions from grey literature were limited to articles focused solely on wood classification initiatives, policies, and regulations, which highlights a critical gap in the existing body of research concerning the integration of classification with valorization processes. Nonetheless, while these documents provide valuable insights into the regulatory frameworks and standards that govern wood classification in different countries in Europe, they fall short of addressing how these classifications can effectively inform and enhance wood valorization strategies. This disconnect emphasizes the necessity for comprehensive research that not only explores the nuances of wood classification but also investigates how these classifications can be leveraged to optimize valorization technologies. Besides, the findings from the grey literature reveal a notable lack of a harmonized wood classification scheme across Europe, particularly regarding policies and regulations. This absence of standardization presents significant challenges for stakeholders in the wood industry, as varying classification criteria can lead to inconsistencies in how wood materials are categorized and managed.

## Discussion

The results of the systematic research review underscore the challenges associated with investigating the valorization of wood waste. Despite the growing interest in sustainable waste management practices, the limited yield of scholarly papers reveals significant obstacles in accessing relevant literature. This limited yield of scholarly papers prompted us to first examine the structure of our query as a potential contributing factor to the challenges encountered in our systematic research review. It appears that the query's construction, particularly its combination of terms across various levels, may inadvertently constrain search results by demanding that all selected concepts coexist within a single document. This necessity can significantly narrow the available literature, as studies may not uniformly employ the same terminologies or may concentrate on particular aspects of valorization technologies. Furthermore, the complexity of using multiple synonyms and related terms can result in a convoluted query that is less likely to align with existing research. Nonetheless, after extensive trials and analyses with different keyword combinations, it can be confidently stated that the refined query represents the best possible approach, yielding the most comprehensive and relevant results for our research objectives. This query serves as a solid starting point, revealing identifiable gaps in the literature—an outcome we anticipated. Although the limited number of relevant papers is noteworthy, the insights gained from the existing literature are invaluable and resonate deeply with our research focus. The query’s design reflects a careful balance between specificity and breadth, yet it inevitably imposes constraints by requiring the co-occurrence of selected terms within single documents.

Moreover, the multidisciplinary nature of the topic complicates the search for relevant literature, as it intersects with several fields, including waste management, material science, engineering, and sustainability studies. Each of these disciplines may utilize distinct terminologies, which could hinder the effectiveness of a rigid query structure. For example, waste management literature might emphasize recycling protocols, while material science could focus on the technical aspects of valorization technologies. Furthermore, the analysis of wood valorization technologies reveals significant disparities in how different databases address relevant keywords, greatly influencing search outcomes and the efficacy of our research. Each database is tailored to specific disciplines, such as forestry, environmental science, and materials science, which leads to keyword variability. For instance, terms like "wood waste" may be represented as "wood residuals" or "wood byproducts" across various databases. This inconsistency in terminology not only complicates the retrieval of relevant studies but also skews the representation of available technologies.

In addition, the systematic review reveals a significant gap between the extensive literature on wood valorization technologies and the actual variety of methods employed in sectors such as construction, bioenergy, and bioproducts. This finding is particularly surprising, as our search did not impose any time limitations, allowing us to capture a broad spectrum of research and innovations in the field. In particular, although there is a wealth of research available, many studies focus primarily on well-known techniques, such as thermochemical and biochemical processes, while neglecting newer technologies that could advance the field. This limited perspective risks sidelining innovative solutions, particularly in mechanical wood valorization processes that include important methods like shredding, grinding, and milling. The absence of these processes in the literature may stem from inadequate search terms or database preferences that favor more complex methodologies, thereby perpetuating a cycle of oversight in effective wood waste management practices. Additionally, the findings from our review indicate a tendency within the academic community to prioritize newer technologies over traditional mechanical processes. These biases limit the exploration of well-established methods, which remain vital for practical applications in wood waste management. To address these gaps, future research should adopt a more inclusive approach to literature reviews, ensuring that diverse wood valorization technologies and methodologies are assessed comprehensively. This would facilitate a richer understanding of existing practices and help identify opportunities for innovation in the industry. Biochemical approaches to wood treatment present promising avenues for enhancing the inherent properties of wood while integrating biological systems into production processes. By leveraging biological mechanisms—such as fungal and enzymatic treatments—these methods optimize resource use and minimize waste, aligning with the principles of a circular economy. Our research underscores the role of pyrolysis and torrefaction in promoting sustainable waste management and resource recovery. Pyrolysis effectively reduces environmental impacts, while torrefaction improves biomass energy density, making it suitable for renewable energy applications. Together, these methods not only valorize wood residues but also advance sustainable energy practices.

To complement these biochemical strategies, advancements in wood sorting and separation technologies are essential for maximizing resource recovery and ensuring efficient material utilization. In the realm of wood sorting and separation technologies, NIR methods stand out for their rapid, non-destructive analysis capabilities, essential for efficient wood species identification and material property assessment. However, the limited scope of current studies raises concerns about the full spectrum of available sorting technologies. To optimize waste management practices, further exploration of alternative techniques, such as machine learning algorithms and laser-induced breakdown spectroscopy, is necessary. These innovations could enhance material identification and sorting efficiency, ultimately contributing to more sustainable practices The integration of these advanced sorting technologies with robust classification decision tools forms a promising approach crucial, as it can significantly improve recycling outcomes and resource recovery efforts.

Finally, as suggested by the case study on wood classification by Pazzaglia and Castellani (2023), future research should delve deeper into recycling processes, optimizing pathways based on wood chemical composition and incorporating comprehensive assessments like Life Cycle Assessment (LCA) and social LCA. Additionally, market analyses tailored to local contexts are crucial for identifying best practices. Bridging the existing gaps is vital for fostering cohesive and efficient wood management practices. A comprehensive understanding of how various classifications affect valorization methods will enhance the selection of appropriate strategies, benefiting both the industry and the environment. A unified framework for wood classification is urgently needed to streamline processes, enhance the effectiveness of valorization initiatives, and align with sustainability goals.

Overall, the study results highlight the critical need for a more comprehensive and inclusive approach to research on wood valorization technologies. By addressing keyword variability, exploring a wider array of methodologies, and fostering collaboration across regulatory frameworks, future studies can unlock new opportunities for resource recovery and contribute to a more sustainable circular economy. Establishing standardized classification systems and embracing innovative technologies will not only facilitate effective wood waste management but also enhance the industry's capacity to meet environmental challenges and promote sustainable practices. The integration of advanced sorting technologies and robust classification decision tools not only addresses the gaps identified in the literature but also aligns with the broader goal of enhancing wood waste valorization. As the systematic review has shown, the challenges in accessing comprehensive literature stem from terminological inconsistencies and a narrow focus on established methods. By expanding our search strategies and embracing innovative sorting technologies, we can improve the identification and classification of wood waste, ultimately leading to more effective recycling and resource recovery efforts. This interconnected approach emphasizes the importance of a cohesive framework that incorporates both traditional and emerging techniques, ensuring that diverse methodologies are considered. Such a framework is essential for fostering innovation and collaboration across disciplines, ultimately contributing to a more sustainable circular economy and improving compliance with varying regulations.

## Conclusion

The findings from this research underscore the significant capacity of wood valorization technologies to enhance sustainable resource management, stimulate economic growth, and safeguard environmental integrity. By effectively transforming wood waste into valuable products, these technologies not only contribute to resource efficiency but also help mitigate environmental impacts associated with waste disposal. This dual benefit is crucial in a time when sustainability is at the forefront of global concerns. However, the research also highlights the necessity of adopting a systematic approach to wood valorization that considers both the opportunities and challenges present in this field. This includes understanding the complexities of various valorization processes, the importance of implementing effective sorting and separation techniques, and the need for harmonized classification schemes. By addressing these multifaceted aspects, the findings advocate for a balanced perspective that not only recognizes the benefits of wood valorization technologies but also actively seeks solutions to the barriers that may hinder their widespread implementation.

## Future perspectives

Looking ahead, it is essential to adopt a holistic approach that extends beyond technological and regulatory considerations to include vital factors such as supply chain management and logistics, as these elements are crucial for optimizing material flow and reducing costs. Financial factors, including cost-effectiveness and investment in innovative technologies, play a significant role in the feasibility and scalability of valorization initiatives. Additionally, integrating Life Cycle Assessment (LCA) and Life Cycle Costing (LCC) methodologies will provide a more comprehensive understanding of the environmental impacts and economic viability of wood valorization processes, enabling stakeholders to make informed decisions that align with sustainability goals. The necessity for a comprehensive wood valorization framework is evident, as integrate various wood valorization technologies should be integrated alongside sorting and separation technologies, while also adhering to wood classification policies and regulations. Such a framework is essential for maximizing the economic and environmental benefits of wood resources, as it would facilitate efficient recycling, reuse, and transformation of wood waste into valuable products. By incorporating advanced sorting and separation technologies, this approach can enhance the quality and purity of recovered materials, ensuring that they meet the necessary standards for various applications. Furthermore, aligning this framework with established wood classification policies is crucial for compliance and to promote sustainable practices across the industry. Ultimately, establishing this comprehensive framework will not only enhance the effectiveness of wood valorization but also contribute to a circular economy, promoting resource efficiency and sustainability within the wood industry and beyond.

## Ethics and consent

Ethical approval and consent were not required for this systematic review.

## Data Availability

No data associated with this article. Zenodo: Wood Waste Valorization and Classification Approaches: A systematic review https://doi.org/10.5281/zenodo.14289244 ( Korba, 2024). Data are available under the terms of the Creative Commons Attribution 4.0 International license (CC-BY 4.0) ( https://creativecommons.org/licenses/by/4.0/).
