# Supplementary material for: Wood Waste Valorization and Classification Approaches: A systematic review
Source: Open Res Eur. 2025 May 6;5:5. Originally published 2025 Jan 10. [Version 2] doi: 10.12688/openreseurope.18862.2 (PMC12118346; doi:10.12688/openreseurope.18862.2)
Supplement: Peer review history

# Reviewer report: Reviewer response for version 2

**DOI:** 10.21956/openreseurope.21988.r53948

**Published:** 2025-05-27

**Authors:** Grzegorz Kowaluk

**Affiliations:** 1 Warsaw University of Life Sciences-SGGW, Warsaw, Poland

Reviewer Summary:

 This article presents a thorough and well-structured systematic literature review (SLR) on technologies and frameworks for the valorization and classification of wood waste, primarily from construction/demolition and the furniture sector. The review is methodologically rigorous, grounded in the PRISMA guidelines, and focused on circular economy objectives. It identifies and analyzes existing technologies and policies, outlines research gaps, and proposes the need for a unified valorization framework.

 1. Is the rationale for, and objectives of, the Systematic Review clearly stated?

 Yes.

 The manuscript provides a compelling background on the environmental and economic importance of valorizing wood waste. The research question is well-articulated, aiming to understand available technologies and criteria for selecting valorization pathways. The rationale for focusing on CDW and furniture waste is clearly justified in terms of waste volumes and lack of standardized classification frameworks.

 2. Are sufficient details of the methods and analysis provided to allow replication by others?

 Yes, with minor reservations.

 The methods are clearly explained, including database selection (ScienceDirect and Scopus), query construction, inclusion/exclusion criteria, and quality assessment. However, the review could benefit from:

 - A brief discussion on the limitations of excluding book chapters and non-English sources, especially given the policy-oriented and multidisciplinary nature of the topic.

 - Additional clarity on how inter-rater reliability was ensured between the two reviewers.

 3. Is the statistical analysis and its interpretation appropriate?

 Not applicable.

 As this is a qualitative SLR, statistical synthesis was not used, and the narrative approach adopted is appropriate. The manuscript adequately synthesizes themes across the reviewed literature.

 4. Are the conclusions drawn adequately supported by the results presented in the review?

 Yes.

 The conclusions are well-aligned with the data presented. The authors emphasize the importance of sorting and classification technologies, gaps in current frameworks, and the need for policy harmonization—all of which are supported by the literature. Recommendations for a holistic, integrated framework are reasonable and reflect the evidence.

 5. Specific strengths

 - Clearly structured and transparent SLR protocol.

 - Thematic classification of findings is well-organized.

 - The manuscript identifies an actionable research and policy gap.

 - Revision addresses previous reviewer concerns (e.g., cascade recovery, grey literature use, mechanical methods).

 6. Specific weaknesses or suggestions for improvement

 - Steam explosion is not mentioned, despite being a relevant thermochemical pretreatment technique for lignocellulosic materials. A brief justification (included in the author response) is appreciated, but the explanation could be placed in the main text.

 - Mechanical recovery processes like shredding or cascade use are briefly acknowledged in version 2, but still warrant more in-depth treatment given their practical importance.

 - NIR hyperspectral technology is discussed, but industry applications (e.g., use by sorting machine manufacturers) could be more explicitly highlighted to connect research and practice.

 - Fungal and bio-based materials research is rapidly evolving. More recent literature could be cited (2023–2024), especially given the high publication rate in this field.

 7. Are the competing interests and funding information appropriately disclosed?

 Yes.

 The manuscript transparently reports EU Horizon funding and potential conflicts of interest (e.g., a reviewer involved in the WOOD2WOOD project).

 8. Overall Recommendation

 Approved with minor reservations.

 The manuscript represents a significant contribution to the field of sustainable resource management and circular economy. With some minor revisions or clarifications (particularly expanding on overlooked valorization methods and updating rapidly evolving subsections), it can serve as a foundational reference for academics, policymakers, and industry practitioners.

Are the rationale for, and objectives of, the Systematic Review clearly stated?

Yes

Is the statistical analysis and its interpretation appropriate?

Not applicable

Are sufficient details of the methods and analysis provided to allow replication by others?

Partly

Are the conclusions drawn adequately supported by the results presented in the review?

Yes

Reviewer Expertise:

Circular economy, bio-based materials, environmental policy, waste valorization technologies.

I confirm that I have read this submission and believe that I have an appropriate level of expertise to confirm that it is of an acceptable scientific standard, however I have significant reservations, as outlined above.

# Reviewer report: Reviewer response for version 2

**DOI:** 10.21956/openreseurope.21988.r53872

**Published:** 2025-05-27

**Authors:** Arnaud Besserer

**Affiliations:** 1 Université de Lorraine, Aiguillettes, France

In the revised version the authors answered to all the comments and made the requested corrections.

Are the rationale for, and objectives of, the Systematic Review clearly stated?

Yes

Is the statistical analysis and its interpretation appropriate?

Not applicable

Are sufficient details of the methods and analysis provided to allow replication by others?

Yes

Are the conclusions drawn adequately supported by the results presented in the review?

Partly

Reviewer Expertise:

Microbiology, Wood sciences, Microscopy, Biobased materials, Bioremediation, green processes

I confirm that I have read this submission and believe that I have an appropriate level of expertise to confirm that it is of an acceptable scientific standard.

# Reviewer report: Reviewer response for version 2

**DOI:** 10.21956/openreseurope.21988.r53871

**Published:** 2025-05-15

**Authors:** Aron Pazzaglia

**Affiliations:** 1 CIRIAF – Interuniversity Research Center, University of Perugia, Perugia, Italy

The new version addresses the suggestions made by the reviewers.

Are the rationale for, and objectives of, the Systematic Review clearly stated?

Yes

Is the statistical analysis and its interpretation appropriate?

Not applicable

Are sufficient details of the methods and analysis provided to allow replication by others?

Yes

Are the conclusions drawn adequately supported by the results presented in the review?

Yes

Reviewer Expertise:

Wood waste management and recycling

I confirm that I have read this submission and believe that I have an appropriate level of expertise to confirm that it is of an acceptable scientific standard.

# Reviewer report: Reviewer response for version 1

**DOI:** 10.21956/openreseurope.20412.r49968

**Published:** 2025-02-12

**Authors:** Arnaud Besserer

**Affiliations:** 1 Université de Lorraine, Aiguillettes, France

This article sets out the basic framework for the recovery of post-consumer wood. It highlights the importance of converting end-of-life wood into a product in order to reduce environmental impact, and the technical and economic feasibility of this approach is discussed at the end of the article.

 The value of implementing decision-making tools for the fate of end-of-life wood products based on the type of post-consumer wood is also aptly addressed. The lack of an overall framework for wood recovery, taking into account classification, sorting and recovery processes, is highlighted as an obstacle to the development of wood recycling. This work highlights the need for a holistic approach to wood waste recovery and the complexity of a multidisciplinary subject.

 The PRISMA approach followed is well explained, but it raises several questions and truncates important points of recent technological advances, some of which are deployed on an industrial scale.

 I don't understand why the book chapters are not of sufficient scientific quality?

 Why isn't steam explosion mentioned as a technology for treating wood waste?

 Hyperspectral NIR technology is already used by sorting machine manufacturers. Why is this not mentioned in the dedicated paragraph?

 Wood classification policies and regulations: what are the sources? Missing references

 The analysis of the effects of "terminological incoherence" is very pertinent: it highlights the consequences of a homogeneity of disciplines that are corollary to the recovery of wood waste.

 The absence of literature on mechanical recovery is probably due to the scope of the study. It would be interesting to consider the cascade recovery of demolition wood and not just its recycling. This would broaden the scope of the study to include reuse and re-utilization, where machining and mechanical recovery are at the heart of research. Similarly, it is well recognized that shredding reduces the value of wood that can be reused. It therefore makes sense to carry out this operation only as a second step. Several studies can be cited:

 Besserer, A., Troilo, S., Girods, P., Rogaume, Y., Brosse, N., 2021. Cascading Recycling of Wood Waste: A Review. Polymers 13, 1752. https://doi.org/10.3390/polym13111752 (Refer 1)

 Schmitz, N., Krause, A., Lüdtke, J., 2023. Critical review on a sustainable circular bio-economy for the forestry sector = Zirkuläre Bioökonomie in der Forst- und Holzwirtschaft für eine nachhaltige Entwicklung - Eine wissenschaftliche Einordnung. Johann Heinrich von Thünen-Institut, DE.(Refer 2)

 Litterature search about fungal based material should be updated as it is a very dynamic research fields with high publication rate.

Are the rationale for, and objectives of, the Systematic Review clearly stated?

Yes

Is the statistical analysis and its interpretation appropriate?

Not applicable

Are sufficient details of the methods and analysis provided to allow replication by others?

Yes

Are the conclusions drawn adequately supported by the results presented in the review?

Partly

Reviewer Expertise:

Microbiology, Wood sciences, Microscopy, Biobased materials, Bioremediation, green processes

I confirm that I have read this submission and believe that I have an appropriate level of expertise to confirm that it is of an acceptable scientific standard, however I have significant reservations, as outlined above.

## References

[1] : Cascading Recycling of Wood Waste: A Review. *Polymers (Basel)* .2021;13(11) : 10.3390/polym13111752 10.3390/polym13111752 34071945 PMC8198968

[2] : Critical review on a sustainable circular bio-economy for the forestry sector.2023;

# Response

**Published:** 2025-04-28

**Authors:** AKRIVI KORBA

**Affiliations:** Institute of Communication and Computer Systems, Athens, Greece

**Comment: The PRISMA approach followed is well explained, but it raises several questions and truncates important points of recent technological advances, some of which are deployed on an industrial scale.** **Response:** Thank you for highlighting this. The PRISMA methodology was adhered to, ensuring a rigorous process for selecting studies. We acknowledge that some recent technological advances, particularly those deployed on an industrial scale, were not covered in depth within the scope of this review. This was largely due to the specific research query's focus on valorization processes and recycling. We appreciate the suggestion and will investigate incorporating more recent technological advancements in future updates of the review. **Comment: Why isn't steam explosion mentioned as a technology for treating wood waste?** **Response:** Steam explosion is not explicitly mentioned in the systematic review due to the structure of the research query and the criteria used to categorize the technologies. The review primarily focuses on valorization processes, which involve converting waste into valuable products. Steam explosion, commonly used to modify wood's physical structure and break down lignocellulosic components, is more of a pretreatment or depolymerization technique rather than a direct valorization method. Additionally, it is often used in processes such as resin or coating removal, which might not align with the inclusion criteria for technologies that directly generate end-products from wood waste. This explains its omission from the final list of technologies included. **Comment: I don't understand why the book chapters are not of sufficient scientific quality.** **Response:** The exclusion of book chapters was not due to their inherent lack of scientific quality but rather due to their typical presentation format. Book chapters, especially those in edited volumes, often provide summaries or overviews of existing research rather than original studies with detailed methodologies. They may not always undergo the same rigorous peer review process or follow the structured presentation of results and methods typical of primary research articles. By focusing on peer-reviewed journal articles, the review aimed to ensure the inclusion of studies with robust, methodologically sound, and reproducible findings that directly contribute to the development of a wood waste valorization framework. **Comment: Hyperspectral NIR technology is already used by sorting machine manufacturers. Why is this not mentioned in the dedicated paragraph?** **Response:** The use of hyperspectral NIR technology is indeed addressed in the dedicated paragraph of the review. If there was any confusion, we apologize for not highlighting it more clearly. Technology is discussed, particularly in the context of its application in sorting and classification processes. **Comment: Wood classification policies and regulations: what are the sources? Missing references.** **Response:** Thank you for pointing this out. *G* *rey literature references have been incorporated into the ‘Wood Classification Policies and Regulations’ section. The corresponding text has been revised to ensure it more accurately reflects the content and insights provided by these sources. Please refer to the revised section below:* Current wood waste classification systems (Wood Recyclers Association, 2023; Environment Agency, 2022) prioritize hazard-based parameters (e.g., treatment types) but lack standardized criteria linking material quality to valorization potential. While UNECE (2023) provides region-specific taxonomies, its failure to address technological suitability for reuse (e.g., structural integrity for cascading) or economic viability of recovery pathways underscores the urgent need for a harmonized international framework. This gap is exacerbated by industry protocols (Timberpak, 2022) that, while rigorous in safety testing, inadvertently fragment markets through inconsistent grading—forcing high-value wood into low-value streams due to incompatible national standards. A unified classification system could resolve these disparities by:

1. Aligning definitions of waste wood grades with their optimal recovery routes (e.g., reuse vs. recycling).
2. Incorporating material performance metrics (e.g., mechanical properties) alongside hazard criteria.
3. Enabling cross-border flows through mutually recognized quality standards.

T *he corresponding grey literature references were added in the 'References' section as well:*

- Environment Agency (2022) Waste Classification Technical Guidance WM3. Bristol: UK Government. Available at: https://assets.publishing.service.gov.uk/media/6152d0b78fa8f5610b9c222b/Waste_classification_technical_guidance_WM3.pdf.
- Timberpak (2022) Wood Waste Classification and Testing Methodology. Leeds: Timberpak Ltd. Available at: https://consult.environment-agency.gov.uk/psc/ls9-0np-timberpak-limited/supporting_documents/Application%20%20Bespoke%20%20NDM%20Response%20%20Wood%20Classification%20and%20Testing%20%20Timberpak%20All%20Sites%20Aug%2022%2007122022.pdf.
- UNECE (2023). Policy Brief: Wood waste and the circular economy. Geneva: United Nations Economic Commission for Europe. Available at: https://unece.org/sites/default/files/2023-02/2228308_E_web_ECE_TIM_DP_91.pdf.
- Wood Recyclers Association (2023) Long-awaited waste wood classification guidance is launched. Brussels: WRA. Available at: https://woodrecyclers.org/long-awaited-waste-wood-classification-guidance-is-launched/

**Comment: The analysis of the effects of "terminological incoherence" is very pertinent: it highlights the consequences of a homogeneity of disciplines that are corollary to the recovery of wood waste.** **Response:** Indeed, inconsistent terminology across different disciplines can lead to misunderstandings and misinterpretations when discussing the recovery of wood waste. This issue can result in fragmented approaches to research and the practical application of findings. Standardizing terminology would promote clearer communication, facilitate interdisciplinary collaboration, and help ensure that stakeholders across various fields have a shared understanding of the processes and technologies involved in wood waste recovery. We appreciate your comment, as it emphasizes the need for greater clarity and consistency in the language used within this field.** *To improve clarity, we have revised the last paragraph in the Discussion section. Please see the changes, which are highlighted in red:*   ''The study underscores an urgent need for harmonized approaches to wood valorization, where fragmented standards and inconsistent terminology currently hinder progress. As grey literature reveals, regulatory disparities across borders create unnecessary complexity for industry stakeholders, while the lack of unified classification systems stifles innovation in waste recovery. Industry reports highlight how these challenges manifest in practice—divergent definitions of wood waste, conflicting compliance requirements, and missed opportunities for scalable recycling solutions. Moving forward, bridging this gap requires a dual focus: aligning policy frameworks with technological advancements (e.g., integrating material science criteria into regulatory categories) and developing shared vocabularies that transcend disciplinary silos. Advanced sorting technologies, from AI-driven identification to spectroscopic analysis, could play a pivotal role in overcoming these barriers, offering precision where traditional methods fall short. Yet technology alone is insufficient without regulatory coherence. A truly effective system must weave together national standards, industry needs, and emerging innovations, transforming wood waste from a logistical challenge into a cornerstone of the circular economy. Such integration would not only streamline compliance but also unlock new pathways for resource recovery, turning today’s fragmentation into tomorrow’s (circular) sustainability success.'' **Comment: The absence of literature on mechanical recovery is probably due to the scope of the study. It would be interesting to consider the cascade recovery of demolition wood and not just its recycling. This would broaden the scope of the study to include reuse and re-utilization, where machining and mechanical recovery are at the heart of research. Similarly, it is well recognized that shredding reduces the value of wood that can be reused. It therefore makes sense to carry out this operation only as a second step.** **Several studies can be cited:**

1. **Besserer, A., Troilo, S., Girods, P., Rogaume, Y., Brosse, N., 2021.** **Cascading Recycling of Wood Waste: A Review. Polymers 13, 1752.**
2. **Schmitz, N., Krause, A., Lüdtke, J., 2023. Critical review on a sustainable circular bioeconomy for the forestry sector.**

**Response:** You are correct that the absence of literature on mechanical recovery is largely due to the specific focus of the research query, which concentrated on valorization and recycling processes. To include cascade recovery, reuse, and mechanical recovery would require altering the structure of the query, thus changing the scope of the systematic review. These topics would indeed broaden the findings, particularly regarding the role of mechanical recovery and machining in prolonging the life of wood waste. As you rightly pointed out, shredding can reduce the quality of wood for reuse, making it more suitable as a secondary recovery step. The studies you cited on cascading recycling and sustainable circular bioeconomy are excellent suggestions and will be considered for future updates of the review. Regarding the reference suggestions, we acknowledge their relevance; however, as those references did not emerge through the structured search and screening process described in the Methods section, their inclusion would fall outside the defined review protocol and potentially compromise the methodological consistency of the study. To address your valuable observations, we have modified the corresponding paragraph in the Discussion section to explicitly acknowledge the gap in mechanical recovery literature and its implications for cascade reuse and value retention (e.g., shredding’s impact on wood quality). Please, see the changes in the text in red: ''In particular, although there is a wealth of research available, many studies focus primarily on well-known techniques, such as thermochemical and biochemical processes, while neglecting newer technologies—as well as established mechanical processes like shredding, grinding, and milling—that could advance the field. This limited perspective risks sidelining innovative solutions, particularly in mechanical wood valorization processes, which are critical for cascade recovery (e.g., reuse and re-utilization) but often overlooked in favor of recycling or energy recovery. The absence of these processes in the literature may stem from inadequate search terms, database preferences favoring complex methodologies, or a narrow scope that excludes mechanical recovery’s role in preserving wood value, thereby perpetuating a cycle of oversight in effective wood waste management practices. Additionally, our review indicates a tendency to prioritize newer technologies over traditional or hybrid methods, despite evidence that shredding and other mechanical treatments can reduce the potential for high-value reuse. To address these gaps, future research should adopt a more inclusive approach to literature reviews, ensuring that cascade recovery pathways and diverse valorization technologies are assessed comprehensively. This would facilitate a richer understanding of practical, value-retaining practices and help identify opportunities for innovation in the industry. Biochemical approaches to wood treatment present promising avenues for enhancing the inherent properties of wood while integrating biological systems into production processe.'' While the core scope of our systematic review remains anchored to the original research query, this revision ensures your critical perspective is formally reflected in our analysis. Future work—guided by your suggestions—could rigorously explore these dimensions through a dedicated review of mechanical recovery’s role in circular wood systems, with adjusted search parameters to capture such studies **Comment: Literature research about fungal-based material should be updated as it is a very dynamic research field with a high publication rate.** **Response:** Fungal-based materials are indeed a rapidly evolving area of research with a high publication rate. However, the results presented in this review are based on the literature identified through the specific research query employed, which captured the most relevant studies available at the time of the review. To capture the latest developments in this dynamic field, it would be beneficial to periodically update the literature search. This would ensure the inclusion of the most recent studies in future revisions of the review.

# Reviewer report: Reviewer response for version 1

**DOI:** 10.21956/openreseurope.20412.r49668

**Published:** 2025-02-03

**Authors:** Aron Pazzaglia

**Affiliations:** 1 CIRIAF – Interuniversity Research Center, University of Perugia, Perugia, Italy

This systematic review explores wood waste valorization and classification systems, delving into ongoing research and existing gaps in the field. The methodology is clear and well-articulated.

 The results identify four main categories in wood waste management: wood valorization processes, wood sorting and separation, wood classification decision tools, and wood classification policies and regulations. The review provides comprehensive knowledge about the current limitations and future perspectives in wood waste management.

 My only suggestion is to be more precise and provide more details regarding the grey literature mentioned in the "Wood Classification Policies and Regulations" section. I have added a reference that could be useful for strengthening this point.

Are the rationale for, and objectives of, the Systematic Review clearly stated?

Yes

Is the statistical analysis and its interpretation appropriate?

Not applicable

Are sufficient details of the methods and analysis provided to allow replication by others?

Yes

Are the conclusions drawn adequately supported by the results presented in the review?

Yes

Reviewer Expertise:

Wood waste management and recycling

I confirm that I have read this submission and believe that I have an appropriate level of expertise to confirm that it is of an acceptable scientific standard.

## References

[1] : WOOD WASTE VALORIZATION IN EUROPE: POLICY FRAMEWORK, CHALLENGES, AND DECISIONAL TOOLS.2023;

# Response

**Published:** 2025-04-28

**Authors:** AKRIVI KORBA

**Affiliations:** Institute of Communication and Computer Systems, Athens, Greece

Thank you for the suggestion. Grey literature references were added as needed and in line with the suggestions received. Regarding this specific reference, it was not included because all sources had to be identified through the systematic review process outlined in the Methods section. As this reference did not appear through the structured search and screening steps, its inclusion would fall outside the defined protocol and compromise the methodological consistency of the review. **Changes in text:** *Grey literature references have been incorporated into the ‘Wood Classification Policies and Regulations’ section. The corresponding text has been revised to ensure it more accurately reflects the content and insights provided by these sources.* *Please refer to the revised section below.* Current wood waste classification systems (Wood Recyclers Association, 2023; Environment Agency, 2022) prioritize hazard-based parameters (e.g., treatment types) but lack standardized criteria linking material quality to valorization potential. While UNECE (2023) provides region-specific taxonomies, its failure to address technological suitability for reuse (e.g., structural integrity for cascading) or economic viability of recovery pathways underscores the urgent need for a harmonized international framework. This gap is exacerbated by industry protocols (Timberpak, 2022) that, while rigorous in safety testing, inadvertently fragment markets through inconsistent grading—forcing high-value wood into low-value streams due to incompatible national standards. A unified classification system could resolve these disparities by:  

1. Aligning definitions of waste wood grades with their optimal recovery routes (e.g., reuse vs. recycling).
2. Incorporating material performance metrics (e.g., mechanical properties) alongside hazard criteria.
3. Enabling cross-border flows through mutually recognized quality standards.

*Grey literature references added to the 'References' section, as well:*

- Environment Agency (2022) Waste Classification Technical Guidance WM3. Bristol: UK Government. Available at: https://assets.publishing.service.gov.uk/media/6152d0b78fa8f5610b9c222b/Waste_classification_technical_guidance_WM3.pdf.
- Timberpak (2022) Wood Waste Classification and Testing Methodology. Leeds: Timberpak Ltd. Available at: https://consult.environment-agency.gov.uk/psc/ls9-0np-timberpak-limited/supporting_documents/Application%20%20Bespoke%20%20NDM%20Response%20%20Wood%20Classification%20and%20Testing%20%20Timberpak%20All%20Sites%20Aug%2022%2007122022.pdf.
- UNECE (2023). Policy Brief: Wood waste and the circular economy. Geneva: United Nations Economic Commission for Europe. Available at: https://unece.org/sites/default/files/2023-02/2228308_E_web_ECE_TIM_DP_91.pdf.
- Wood Recyclers Association (2023) Long-awaited waste wood classification guidance is launched. Brussels: WRA. Available at: https://woodrecyclers.org/long-awaited-waste-wood-classification-guidance-is-launched/
